# Ultrasonographic Assessment of the Normal Femoral Articular Cartilage of the Knee Joint: Comparison with 3D MRI

**DOI:** 10.1155/2021/9978819

**Published:** 2021-08-18

**Authors:** Kyösti Kauppinen, Victor Casula, Štefan Zbýň, Roberto Blanco Sequeiros, Simo S. Saarakkala, Mika T. Nevalainen

**Affiliations:** ^1^Medical Research Center Oulu, University of Oulu, P.O. Box 8000, Oulu, Finland; ^2^Research Unit of Medical Imaging, Physics and Technology, Faculty of Medicine, University of Oulu, P.O. Box 5000, FI-90014 Oulu, Finland; ^3^Department of Radiology, Turku University Hospital, P.O. Box 52, 20521 Turku, Finland; ^4^Center for Magnetic Resonance Research, Department of Radiology, University of Minnesota, 6th St SE, Minneapolis, MN 55455, USA; ^5^Department of Diagnostic Radiology, Oulu University Hospital, P.O. Box 50, 90029 Oulu, Finland

## Abstract

**Objective:**

Ultrasonography (US) has a promising role in evaluating the knee joint, but capability to visualize the femoral articular cartilage needs systematic evaluation. We measured the extent of this acoustic window by comparing standardized US images with the corresponding MRI views of the femoral cartilage.

**Design:**

Ten healthy volunteers without knee pathology underwent systematic US and MRI evaluation of both knees. The femoral cartilage was assessed on the oblique transverse axial plane with US and with 3D MRI. The acoustic window on US was compared to the corresponding views of the femoral sulcus and both condyles on MRI. The mean imaging coverage of the femoral cartilage and the cartilage thickness measurements on US and MRI were compared.

**Results:**

Mean imaging coverage of the cartilage of the medial femoral condyle was 66% (range 54%–80%) and on the lateral femoral condyle 37% (range 25%–51%) compared with MRI. Mean cartilage thickness measurement in the femoral sulcus was 3.17 mm with US and 3.61 mm with MRI (14.0% difference). The corresponding measurements in the medial femoral condyle were 1.95 mm with US and 2.35 mm with MRI (21.0% difference), and in the lateral femoral condyle, they were 2.17 mm and 2.73 mm (25.6% difference), respectively.

**Conclusion:**

Two-thirds of the articular cartilage of the medial femoral condyle, and one-third in the lateral femoral condyle, can be assessed with US. The cartilage thickness measurements seem to be underestimated by US. These results show promise for the evaluation of the weight-bearing cartilage of the medial femoral condyle with US.

## 1. Introduction

Ultrasonography (US) examination of the knee joint is a good imaging technique to assess the superficial structures of the knee. As compared to the conventional radiography, US allows the evaluation of the soft tissues in addition to the bony contour of the knee; accordingly, effusion, synovial hypertrophy, meniscal extrusion, osteophytes and part of the femoral cartilage can be assessed with US [1]. Commonly, US examination is performed with the patient in the supine position with knee fully extended and, subsequently, with 90° to 120° flexion allowing the visualization of the femoral cartilage. Using this maneuver, the cartilage of the superior aspect of the femoral sulcus and both femoral condyles can be evaluated to some extent. However, it remains unclear whether the actual weight-bearing cartilage of the tibiofemoral joint is visualized. Many studies have shown that evaluation of the medial compartment of the knee joint is feasible via US, whereas on the lateral compartment, the evaluation is more limited [2–7]. Recently, Cao et al. introduced a longitudinal knee US scanning technique suggesting comparable results with routine MRI concerning knee cartilage defects [8]. Despite previous research, it is yet unknown which portion of the femoral cartilage is visualized on the US examination. Therefore, the purpose of this study was to assess the acoustic window on US examination of the knee joint by comparing the US and corresponding high-resolution MRI views of the femoral cartilage on knees of healthy volunteers. Moreover, comparison of the cartilage thicknesses measured with these imaging modalities was performed.

## 2. Materials and Methods

### 2.1. Study Population

Ten healthy volunteers (five males and five females), without knee symptoms, previous knee trauma, or any clinical signs of osteoarthritis or any other cartilage pathology were enrolled into this study. The mean body mass index (BMI) was 23.1 kg/m^2^ (range 19.1–26.3 kg/m^2^), and the mean age was 31 years (range 22–39 years). Both knees of the volunteers were studied, and the total number of examined knees was twenty.

### 2.2. Imaging Technique and Analysis

#### 2.2.1. Ultrasonography

US imaging was performed using the GE LOGIC Expert S7 ultrasound device (GE Healthcare, Milwaukee, WI, USA) with a 15 MHz linear transducer (type ML6–15). B-mode imaging settings were kept constant for each patient, and the focus was set at the level of the region of interest. A standard US of the knee^1^ assessing femoral cartilage was performed by a single radiologist (M.N.) with five years of experience in musculoskeletal US. The knee was scanned with the patient in the supine position with the knee flexed to 90° to evaluate the femoral cartilage: transverse axial US images perpendicular to the cartilage surface were acquired from the femoral sulcus to assess the patellofemoral joint and from medial and lateral femoral condyles infrapatellarly to assess the tibiofemoral joint. A goniometer was applied to standardize the knee flexion angle to 90°.

#### 2.2.2. MRI

**Figure 1:**
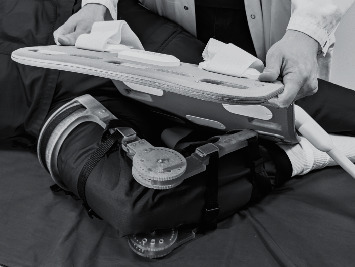
Positioning of the bent knee for the MRI scan with the in-house MRI-compatible 3D-printed brace. The 18-channel body matrix coil was placed over the knee joint.

Following the US examination, MRI was performed on a 3T system (Skyra, Siemens Healthcare, Erlangen, Germany) using a 32-channel spine matrix coil integrated into the scanner table and an 18-channel body matrix coil. An in-house MRI-compatible 3D-printed brace was used to keep the knee flexion angle constant during MR examination. The subject was scanned lying on the same side as the knee of interest with the knee at 90° flexion (Figure 1). The imaging protocol included a 3D T1-weighted VIBE sequence (repetition time (TR)/ccho time (TE) = 4.21/1.34 ms, acquisition matrix = 320 × 260, FOV = 450 ×365 mm^2^, number of slices = 144, slice thickness = 1.5 mm, flip angle = 9 degrees, echo train length = 2, bandwidth = 745 Hz/pixel, and acquisition time = 19 s) and a 3D DESS sequence (TR/TE = 14.1/5.0 ms, acquisition matrix =256 × 256, FOV = 160 × 160 mm^2^, number of slices = 120, slice thickness = 0.6 mm, flip angle = 25 degrees, echo train length = 2, bandwidth = 250 Hz/pixel, and acquisition time = 6 min 41 s). The T1 sequence was applied to verify the flexion angle between the femur and tibia during the MRI exam. Flexion angle between 80 to 100 degrees was accepted in this study. The mean flexion angle was 87.6 degrees (standard deviation (SD) 4.76, range 82–98). The DESS sequence was used for the cartilage measurements.

#### 2.2.3. Image Comparison and Analysis

**Figure 2:**
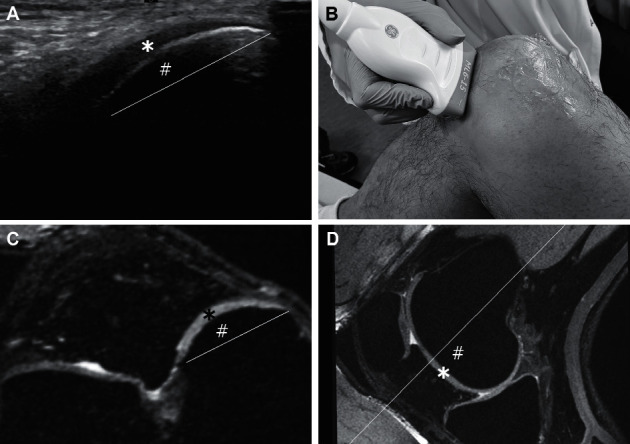
Oblique transverse infrapatellar ultrasonographic image of the medial femoral condyle cartilage (a) was compared to the MR image in the MPR mode (c) using a matching imaging plane. Cartilage thickness was measured from both images (a, c). Condyle width was calculated from MR image (line in (c)) and visible condyle width from US (line in (a)). Sagittal view (d) from the level of the medial part of the patella and lateral part of the medial femoral condyle shows the imaging plane (line in (d)) corresponding to the MR image (c) and also corresponds to the US probe position (b) and US image (a). (^*∗*^) represents the medial femoral cartilage, and (^#^) represents the medial femoral condyle.

**Figure 3:**
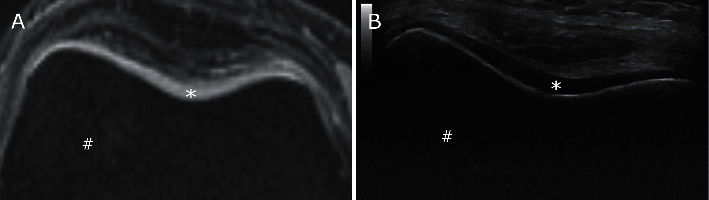
Suprapatellar MR image of the femoral sulcus cartilage (a) and the corresponding US image (b). These images show that the bone (^#^) to cartilage (^*∗*^) interface and, in particular, the cartilage to anterior soft tissue interface are slightly blurred on MRI (a) as compared to US (b) where these interfaces are clearly defined. This could explain the overestimation of cartilage thickness in MRI measurements. Cartilage thickness of the central sulcus (^*∗*^) was measured at 3.0 mm in MRI and 2.4 mm in US image.

Since the stack of isotropic 3D DESS images was obtained for each knee, we were able to compare the US views adaptively to the MR images. First, the 3D image stacks were visualized in multiplanar imaging (MPR) mode in clinical imaging software neaView Radiology (neagen Oy, Helsinki, Finland). Subsequently, starting with common axial, coronal, and sagittal views, the axial view was rotated three dimensionally to achieve the view corresponding to the respective standard US imaging plane. After that, a direct visual comparison was performed to ensure that the MR image corresponded to the imaging plane used in US examination. Both the lateral and medial condyle widths were measured using ImageJ2 software [9]. Since imaging artefacts impede viewing the condyles in their entirety with US, the width was measured from the outer corner of the condyle to the innermost point where the condylar cartilage was visible and clearly defined. The mean cartilage coverage on US was then calculated as the ratio between the condyle width measured on US and the condyle width measured on MRI and expressed in percent. Furthermore, the cartilage thickness was measured from the middle of the sulcus and both medial and lateral edges of the sulcus (superior aspect of the condyles) to assess the patellofemoral joint and from the medial and lateral femoral condyles infrapatellarly to assess the tibiofemoral joint. Figures 2 and 3 demonstrate the comparison between US and MRI. One reader (K.K.) performed the measurements.

### 2.3. Statistical Analysis

The difference in the US cartilage coverage between male and female subjects was evaluated using the paired *t* test. The difference in cartilage thicknesses measured by US and MRI was assessed using Student's *t* test. SPSS 23.0 was used to perform the statistical analyses.

## 3. Results

**Figure 4:**
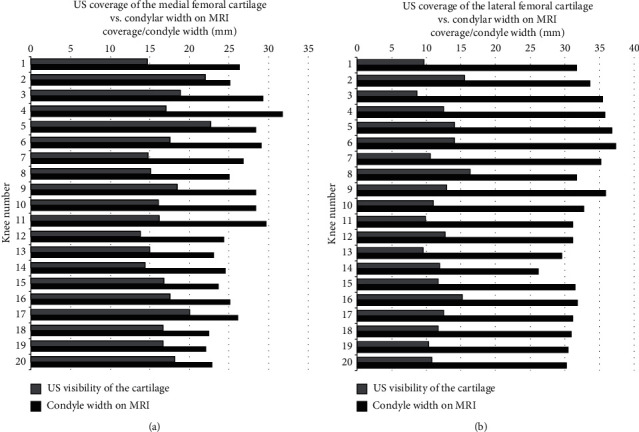
Relative cartilage coverage on US as compared to condyle width measured by MRI. For each knee, the width (mm) of the medial (a) and lateral (b) condyle on MRI (solid bars) and the width (mm) of the visualized cartilage on US (bars with diagonal lines) are given.

In the twenty knees, we assessed the mean cartilage coverage on the US examination was 66% (range 54%–80%) for the medial femoral condyle and 37% (range 25%–51%) for the lateral femoral condyle, when compared to the visualization of condylar cartilage width on MRI. No statistical difference between male and female patients regarding to the US cartilage coverage was observed. On MRI, the mean width of the medial condyle was 26.2 mm (SD 2.67 mm), and on US, the mean width of the visible condyle cartilage was 17.1 mm (SD 2.34 mm). On the lateral condyle, the mean widths were 32.5 mm (SD 2.74 mm) and 12.1 mm (SD 2.07 mm) for MRI and US, respectively.  Figure 4 shows the proportional cartilage coverage on medial and lateral femoral condyles between US and MRI.

The mean cartilage thickness on MR images in the patellofemoral joint cartilage was measured at 3.61 mm in the sulcus, 2.35 mm on the medial edge, and 2.44 mm on the lateral edge of the sulcus. On corresponding US images, the mean cartilage thickness in the sulcus, medial edge, and lateral edge of the sulcus was measured at 3.17 mm, 2.04 mm, and 2.08 mm, respectively. For the tibiofemoral joint, mean cartilage thickness on MRI was 2.35 mm for the medial condyle and 2.73 mm for the lateral condyle. On US, the mean cartilage thickness in the medial and lateral condyle was 1.95 mm and 2.17 mm, respectively. The mean MRI cartilage thickness measurements were 0.315–0.557 mm greater than the measurements on US (Table 1).

## 4. Discussion

Our comparison between US and high-resolution MRI clearly demonstrates that the acoustic window is sufficient to cover the majority of the central portion of the medial femoral cartilage on the knee US. In the present study, the mean coverage of the medial femoral condyle in 20 knees was 66% on US examination. Importantly, in all our subjects, the maximal visibility was over 50% of the width of the medial condylar cartilage surface. This suggests that the clinically relevant central, i.e., weight-bearing, part of the medial femoral condyle is accessible via US evaluation. In previous studies, the issue of acoustic window to the femoral cartilage surface has been omitted, which has restricted the use of US in clinical settings for evaluation of the femoral cartilage. To our knowledge, this is the first study to compare US to MRI regarding to the visibility of femoral condylar cartilages.

It has been shown that the general visibility of the knee joint on US is better medially than laterally [2–7]. This can be explained by the knee anatomy. When the knee joint is flexed, the patella moves laterally and the medial edge of the tibia moves anteriorly exposing the medial femoral condyle. The central portion of the medial femoral cartilage is of clinical interest since it is the main weight-bearing region [10], thus being most prone to cartilage wearing [11–13]. If only the suprapatellar US scanning technique is used, the cartilage of the patellofemoral joint is seen, but virtually, no view to tibiofemoral joint is established, especially to the weight-bearing region. In order to gain the view to the tibiofemoral joint, the knee must be scanned in at least 90° flexion to assess the femoral condyles. Accordingly, on US, the medial infrapatellar view corresponds to the medial weight-bearing region of the cartilage, as presented by Koo et al. [14]. Cao et al. recently presented interesting results on the assessment of the tibiofemoral joint cartilage by MRI and arthroscopy [8]. However, neither Koo et al. nor Cao et al. addressed the US coverage of the condylar surface in their studies.

In this study, the femoral cartilage thicknesses were consistently lower in US than in MRI. In our image comparisons, we found that the bone to cartilage interface and, in particular, the cartilage to anterior soft tissue interface were slightly blurred on MRI compared to US where these interfaces were clearly defined. This could cause measurement error and, at least in part, may explain the overestimation of cartilage thickness in our MRI measurements.

Yoon et al. demonstrated good agreement between US and MRI measuring the maximum thicknesses of the femoral cartilage. However, the minimum thicknesses assessed on US were significantly overestimated as compared with MRI [15]. The review paper by Torp-Pedersen et al. reported that most studies assessing the articular cartilage with US underestimated the thicknesses. The authors proposed this may be due to excluding the leading interface as part of the cartilage and not correcting for the higher sound speed in cartilage [16]. Therefore, it is plausible that US underestimates the thickness of the femoral cartilage. For instance, two studies in healthy children reported cartilage thicknesses 0.5 to 1.1 mm thinner when using US as compared to evaluation using MRI [17, 18]. Supporting this, Schmitz et al. found that US thicknesses were positively correlated with MRI, but poor absolute agreement existed between the measurements obtained from these two imaging modalities, with US thicknesses 1.9 to 2.8 mm smaller than those on MRI [19]. On the microscopic level, the US assessment of cartilage morphology seems to correlate with the histological grade [20, 21]. Interestingly, for the thicker cartilages, the measured thickness was significantly thinner on US than on histology. Maeguchi et al. concluded that US grading and thickness measurements are sufficiently reliable to reflect the histological status [21]. Ultimately, we are proposing that US measures may be a relative indicator of cartilage thickness but should not be used as an absolute measure.

There are limitations in this study. First, the study population consisted only of young and healthy adults without knee symptoms or any clinical signs of osteoarthritis or any other cartilage pathology. When studying patients with osteoarthritis and cartilage defects, the visibility can be inferior. Second, in our study, we performed the US examination with the knee in 90° flexion, although some studies have utilized higher flexion angle up to 135°. However, it remains obscure whether greater flexion angle actually improves the acoustic window on the femoral cartilage. Furthermore, we did not mark the precise position of the US probe, but meticulously extrapolated it on the MR images. Third, we only applied the transverse scanning of the femoral condyles, whereas sagittal scanning has been also shown to be effective. Fourth, the cartilage thickness measures were performed only once by one reader. Fifth, although we assessed the condylar cartilage on multiple transverse axial planes, to evaluate the true coverage of the cartilage on US, area measurements should be applied. Finally, even if it is feasible to use US to evaluate the femoral cartilage of the knee joint, there is no acoustic window to the tibial cartilage, which weakens the overall value of US examination.

## 5. Conclusions

A significant portion of the cartilage of the medial femoral condyle can be visualized with US. On the lateral femoral condyle, the US evaluation may not be sufficient enough for clinical work. Finally, the cartilage thickness seems to be overestimated by MRI as compared to US.

## Figures and Tables

**Table 1:** Mean cartilage thicknesses measured on MR and US images at the patellofemoral joint suprapatellarly (center of the sulcus and the medial and lateral edge of the sulcus) and at the tibiofemoral joint infrapatellarly (medial and lateral femoral condyles).

*Patellofemoral joint (suprapatellar view)*							
Tibial surface							
		Lateral		Sulcus		Medial	
		Mean	Range	Mean	Range	Mean	Range
	MRI (mm)	2.44	1.87–3.43	3.61	2.41–5.00	2.35	1.57–4.05
	US (mm)	2.08	1.40–2.68	3.17	1.82–4.30	2.04	1.28–3.33
MRI/US difference %		17.8%		14.0%		15.5%	

*Tibiofemoral joint (infrapatellar view)*							
Femoral condylar cartilage							
		Lateral		Medial			
		Mean	Range	Mean	Range		
	MRI (mm)	2.73	1.72–4.40	2.35	1.73–3.99		
	US (mm)	2.17	1.50–2.82	1.95	1.31–2.84		
MRI/US difference %		25.6%		21.0%			

## Data Availability

Data can be made available on request.
